# Advanced Glycation End Products Increase MDM2 Expression via Transcription Factor KLF5

**DOI:** 10.1155/2018/3274084

**Published:** 2018-09-09

**Authors:** Pu Wang, Yu Cheng Lu, Yuan Fei Li, Lan Wang, Shao Chin Lee

**Affiliations:** ^1^School of Life Sciences, Shanxi University, Taiyuan, Shanxi 030006, China; ^2^Department of Oncology, The First Clinical Hospital of Shanxi Medical University, Taiyuan, Shanxi 030006, China; ^3^Department of Bological Science, School of Life Sciences, Jiangsu Normal University, Xuzhou, Jiangsu 221000, China

## Abstract

Type 2 diabetes increases the risk for all-site cancers including colon cancer. Diabetic patients present typical pathophysiological features including an increased level of advanced glycation end products (AGEs), which comes from a series of nonenzymatic reactions between sugars and biological macromolecules, positively associated with the occurrence of diabetic complications. MDM2 is an oncogene implicated in cancer development. The present study investigated whether diabetes promoted MDM2 expression in colon cells and the underlying mechanisms. Our results showed that AGE increased the protein level of MDM2 in a cell model and promoted binding between MDM2 and Rb as well as p53, which led to degradation of Rb and p53. KLF5 was able to bind to the regulatory sequence of the MDM2 gene, and knockdown of the KLF5 protein level inhibited the AGE-triggered MDM2 overexpression, which indicated that KLF5 was the transcription factor for MDM2. In a mouse model of diabetes, we found that AGE level was increased in serum. The protein levels of both KLF5 and MDM2 were increased. KLF5 was able to bind to the regulatory sequence of the MDM2 gene. In conclusion, our results suggest that diabetes increases the level of AGE which enhances the expression of MDM2 via transcription factor KLF5 in colon cells. MDM2 overexpression is a candidate biological link between type 2 diabetes and colon cancer development.

## 1. Introduction

Type 2 diabetes is a prevalent endocrine disease worldwide, which causes tremendous economic and health burden [1]. Epidemiologic research suggests that people who suffered from this diabetes have a significantly increased risk for many types of cancer such as colon cancer, endometrial cancer, and breast cancer [2]. For colon cancer, in particular, a 1.5- to 3.0-fold increase in cancer risk is observed [3–5]. Nevertheless, the biological links between type 2 diabetes and cancer remain unknown. Common pathophysiological factors in diabetes, hyperinsulinemia, hyperglycemia, increased level of saturated fatty acids, and advanced glycation end-products (AGEs) [2], are associated with increased cancer risk. However, the underlying molecular mechanisms are to be explored.

AGEs are a complex and heterogeneous group of compounds which comes from a series of nonenzymatic reactions between sugars and biological macromolecules (proteins, lipids, and nucleic acid) [6]. AGEs have been shown to accumulate in various tissues under diabetic conditions, and they participate in the development of diabetic vascular complications (nephropathy, retinopathy, and atherosclerosis) and nondiabetic disease such as senescence and tumours [7]. Glycosylated hemoglobin A1 has been used as an important indicator for diabetes diagnosis and treatment outcome. There is evidence of an association between AGEs and cancer development; epidemiological data demonstrate that the serum concentration of glyceraldehyde-derived AGEs in patients with colorectal cancer is increased and is closely related to an increased risk of rectal cancer [8]. Experiments *in vitro* show that AGEs can promote migration and invasion of breast and colon cancers through the ERK-MMP pathway; moreover, AGEs can enhance colon formation, as observed in soft agar colon formation assay [9, 10]. When BSA drove AGEs are administered ip, they are able to promote the liver metastasis of human colorectal cancer [11].

MDM2 is an oncogene, the amplification and overexpression of which are linked to progression and poor prognosis of different cancers. It can bind directly to cancer suppressors p53 and Rb to promote their inactivation and/or degradation [12].

In clinical samples, increase in MDM2 gene amplification and cytoplasmic expression are observed in colon cancer, which is associated with advanced cancer staging [13]. However, many tumours exhibit high MDM2 and MDMX protein levels without increased copy number [14]. Thus, transcriptional regulation of MDM2 expression in different tissues appears to be the mechanism for increased MDM2 expression [14]. Several transcription factors have been found in MDM2 transcriptional regulation, which include SP1, p53, NF-*κ*B, and KLF6 [15–19]. MDM2 is expressed at different levels in different tissues without known reasons and mechanisms; trigger- or tissue-specific transcriptional factors have not been documented [14].

Kruppel-like factor 5 (KLF5) belongs to the family of Kruppel-like transcription factors, of which 17 members have been identified to date. Members of this family have been implicated in an extensive array of biological processes including embryonic development, control of cellular proliferation and differentiation, and stress response [20]. KLF5 is expressed in the reproductive organs, pancreas, prostate, skeletal muscle, and lung [21]. However, the highest level of its expression is found in intestinal epithelial cells [22]. Studies *in vivo* have demonstrated essential roles of this protein in various biological processes. KLF5 can function as a transcriptional activator or repressor, a promoter or inhibitor of cell growth and survival, and either an oncogene or tumor suppressor, depending on the cellular and genetic context in which it operates [23]. More importantly, evidence suggests that KLF5 plays an important role in intestinal tumorigenesis. In LGR5^+^ stem cells, which contain an oncogenic mutant *β*-catenin, the production of lethal adenomas and carcinomas is completely inhibited by KLF5 deletion [24].

KLF family members are closely related to Sp1 transcription factors but are distinguished by a unique pattern of three cysteine-2/histidine-2 zinc finger motifs separated by seven conserved amino acids at the carboxy terminal of the proteins [25]. Both KLF5 and SP1 bind to similar elements within GC-rich promoter sequences of target genes. Existing data show that SP1 targets at the MDM2 gene basally or under different stimuli [26]. Differential transcriptional activities between SP1 and KLF5 have not been well documented.

From the above, we have a hypothesis that AGEs could promote colon cancer development through active KLF5.

## 2. Materials and Methods

### 2.1. Cell Culture

HCT116 (human colon cancer cell) cells were kindly provided by Dr. B. Vogelstein of the Johns Hopkins University School of Medicine, which were maintained in DMEM (low glucose, 5 mM) supplemented with 50 U/ml penicillin, 50 *μ*g/ml streptomycin, and 10% (*v*/*v*) fetal calf serum. The culture medium and reagents for the colon cancer cells were purchased from Gibco (Beijing, China).

### 2.2. Chemicals and Antibodies

Chemicals were purchased from Sigma (St. Louis, MO, USA). Anti-KLF5 antibody (sc-398470, mice, 1 : 1000) was purchased from Santa Cruz (California, USA). Rabbit antibodies against p53 (D120082, 1 : 500), Rb (D221069, 1 : 500), p-Rb (D151284, 1 : 500), MDM2 (D155246, 1 : 500), SP1 (D161137, 1 : 500), actin (D110001,1 : 1000), anti-histone 2A antibody (D151717, 1 : 1000), and anti-BSA (D120272, 1 : 200) were purchased from Sangon (Shanghai, China). Anti-rabbit fluorescent antibody flux 488 (number 4412, Goat, 1 : 1000) was purchased from Cell Signaling. CHIP Kit (P2078) and Cell Cycle Detection kit (C1052) were purchased from Beyotime (Shanghai, China).

### 2.3. Preparation of AGEs

50 mg/ml BSA (Sigma, USA) was incubated with 0.5 mmol/l D-glucose (Sigma, USA) for 8 weeks at 37°C. The unincorporated sugar was removed by dialysis against 0.2 mmol/l PBS (pH 7.4). 50 mg/ml nonglycated BSA was incubated without D-glucose as a negative control. The AGEs preparations were scanned for fluorescent intensity using a fluorospectrophotometer (SpectraMax M5, USA). AGEs had fluorescent peak at excitation wavelength of 370 nm and slit 2 nm. Data were processed using GraphPad Prism 6.

### 2.4. Flow Cytometric Analysis of AGEs Cell-Binding Assay

HCT116 cells seeded on 6-well plates were treated with 50 *μ*g/ml AGEs at 37°C for 4 hours. Cells were collected by centrifugation at 1000*g* for 5 min, washed with ice-cold PBS, and then fixed with 70% cold ethanol and stored at 4°C for 24 h. Cells were centrifuged again, washed with cold PBS twice, and incubated with anti-BSA antibody for 1 hour at room temperature, and then cells were washed three times with ice-cold PBS followed by FITC-conjugated secondary antibody in 1x PBS for 1 hour at room temperature in the dark, then cells were washed again and measured by flow cytometry. Data were analyzed by FlowJo 7.6.

### 2.5. Western Blot Analysis

The cultured HCT116 cells and mouse colon tissues were lysed in RIPA buffer. Proteins were separated by polyacrylamide gel electrophoresis and transferred onto PVDF membrane. After blocking for 1 hour at room temperature with TBST containing 0.05% (*v*/*v*), Tween-20, and 5% (*w*/*v*) nonfat milk, the membranes were incubated with primary antibodies overnight at 4°C, followed by washes with TBST containing 0.05% Tween-20. The membranes were then incubated with a horseradish peroxidase-conjugated secondary antibody for 1 hour at room temperature. ECL reagents (number 32106, Thermo Biosciences) were used to visualize the protein bands, which were captured on an X-ray film.

### 2.6. Knockdown of Protein Level

The predesigned siRNA oligonucleotides (Sangon Technology, Shanghai, China) were as follows:

1. KLF5, 5′-AAAGUAUAGACGAGACAGUGC-3′ (sense) and GCCTGTCTCGTCTTCTTT (antisense)
2. SP1, 5′AACAGCGTTTCTGCAGCTACC-3′(sense) and GGTAGCTGCAGAAACGCTGTT (antisense)
3. RAGE, 5′-GCCGGAAAUUGUGAAUCCUTT-3′ (sense) and AAGGTTCCTTTCCGGC (antisense)

HCT116 cells (5 × 10^4^ cells per well) were seeded in 6-well plates and cultured for 24 hours and then were transfected with 200 nM SiRNA oligonucleotides using Lipofectamine 2000 transfection reagent (11668-019, Invitrogen), according to the manufacturer's instructions. Transfection efficiency was evaluated by Western blot analysis 24 hours after transfection.

### 2.7. Immunoprecipitation

Cultured HCT116 cells were harvested under nondenaturing conditions, washed by ice-cold PBS for 3 times, lysed in 0.5 ml ice-cold cell lysis buffer, and centrifuged. The supernatant was collected to a new tube and incubated with 20 *μ*l Protein G Plus/Protein A agarose (IP05 Millipore) with gentle shaking for 2 hours at 4°C. Protein G Plus/Protein A agarose was then removed by centrifuge for 10 min at 4°C, and the supernatant was incubated with primary antibody overnight at 4°C with gentle shaking. Afterwards, 30 *μ*l Protein G Plus/Protein A agarose were added and incubated under gentle shaking for 4 hours at 4°C. Finally, Protein G Plus/Protein A agarose was collected by centrifuge followed by 2x loading buffer resuspension and heating at 100°C and microcentrifuge for 1 minute at 14000*g*.

### 2.8. Chromatin Immunoprecipitation

Cultured HCT116 cells were washed and cross-linked using 1% formaldehyde for 20 min, and mouse colon tissues were cross-linked using 1% formaldehyde for 30 min and homogenized. After stopping cross-linking by addition of 0.1 mol/l glycine, cell lysates were sonicated and centrifuged. Immunoprecipitations were performed using the KLF5 antibody in the presence of BSA/salmon sperm DNA and a 50% slurry of protein A agarose beads. Input and immunoprecipitates were washed and eluted and then incubated for 2 h at 42°C in the presence of proteinase K followed by 6 h at 65°C to reverse the formaldehyde cross-linking. DNA fragments were recovered by phenol/chloroform extraction and ethanol precipitation. The related fragments on promoters of MDM2 were amplified by reverse transcription PCR. DNA bands on agarose gels were detected on a UV transilluminator, and graphs were reverse treated by FluorChem HD2 (Alpha Innotech 1.3.0.7). Primers used were as follows:

- Human primer 1 GGAGTGTCACAGCGCCAAA (forward) and CAATTGGGTCCGGGGCTC (reverse)
- Human primer 2 TAAAAGCGCAGAGTAACCGCT (forward) and CGCTGGAGTTGTACCCAAATG (reverse)
- Human primer 3 GTGGACACTGAGTCATACTGCT (forward) and AAAAGAAGGGTCAGAAATGAAGGC (reverse)
- Mouse primer 1 ATTGCGGTTTCGAGCGGTAA (forward) and GCCGCCTCCTGGACCAATA (reverse)
- Mouse primer 2 TGTGGCGTGAGTGACTGAAA (forward) and AAGACTTTAAAAATAGGCAAGGTGA (reverse)

### 2.9. Diabetic Mouse Model

Diabetes was induced in ICR mice by consecutive injection of 50 mg/kg streptozotocin (STZ) (0.05 mol/l sodium citrate, pH 5.5) for 5 days after an 8 h fast. Animals with fasting blood glucose >14 mg/dl were considered to be diabetic. After 8 weeks of injections, blood samples were collected by removing the eyeball; latter, mice were killed by dislocation of the cervical spine. Blood routine examinations were analyzed at the Hospital of Shanxi University by flow cytometry. All *in vivo* procedures were performed according to the National Institutes of Health Guide for the Care and Use of Laboratory animals. Animals were maintained at a barrier area of the laboratory animal center, and padding was changed every day. Blood AGEs were detected by fluorescence quantification at excitation wavelength 370 nm and emission wavelength was 440 nm. The fluorescent intensity was aligned by serum protein concentration.

### 2.10. Statistical Analysis

All of the experiments were performed in triplicate. The data in figures were expressed as mean ± SD, the data of quantified blot bands were expressed as mean (fold) ± SD, and nonparametric test was performed to compare the difference between two groups. One-way ANOVA was performed to show the time-dependent effects observed in the study. The statistical analysis software package SPSS21 was employed for statistical comparisons. A *p*value < 0.05 was considered as statistically significant.

### 2.11. Experimental Design

Firstly, we prepared the AGEs using the BSA-glucose incubation method. Flow cytometry was performed to verify its ability of binding the HCT116 cancer cell line. Then, we analyzed the protein levels of p53, Rb, p-Rb, and MDM2 by Western blot. Finally, we examined whether KLF5 was the transcription for MDM2. Candidate sequences of KLF5 binding to the human MDM2 gene regulatory sequence were predicted in the JASPAR database. Its binding ability was also verified by chromatin immunoprecipitation in the HCT116 cell line. We also quantified the protein level of klf5 and MDM2 and their binding in the colon tissues of healthy and diabetic mice, to show that their expressions and binding were also elevated in colon tissues in diabetes.

## 3. Results

### 3.1. BSA-AGEs Quality and Its Cell Binding

**Figure 1:**
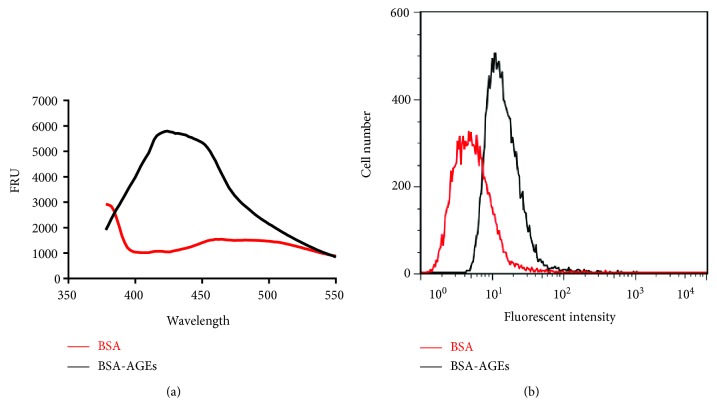
Quanlity assessment of BSA-AGEs and their binding to cells. (a) Full-wavelength scanning was performed to analyze the fluorescent density of quanlity of the prepared AGEs, which indicated the aunlity of the preparation. Excitation wavelength was 370 nm. (b) Cells were treated with AGEs for 4 hours and stained with indirect immunofluorescence staining against BSA and then measured by flow cytometry, which indicated the cell binding of AGE. Every experiment was repeated at least three times.

Wavelength spectrum scanning showed that the prepared BSA-AGEs had an obvious absorption peak at the wavelength between 400 and 450 nm (Figure 1(a)). There was a significant increase (6.1 ± 0.8, *p* < 0.01) in the area under the curve. Next, we investigated the cell-binding ability of the prepared BAS-AGEs using flow cytometry analysis. The results showed that after treatment with BSA-AGEs, cell fluorescence intensity was significantly increased, compared with that in the cells treated with BSA alone (23.1 ± 3.1 versus 7.81 ± 0.65; *p* < 0.01) (Figure 1(b)). Results from MTT assay showed that AGEs did not inhibit cell viability from the concentrations from 50 to 300 *μ*g/ml (figure not shown). We used the concentration of 50 *μ*g/ml in all the experimental treatments, since the cell appeared to psroliferate well at this concentration of BSA-AGEs.

### 3.2. BSA-AGEs Enhances the Expression of MDM2 and Phosphorylation of Rb and Decreases the Protein Levels of Rb and p53

**Figure 2:**
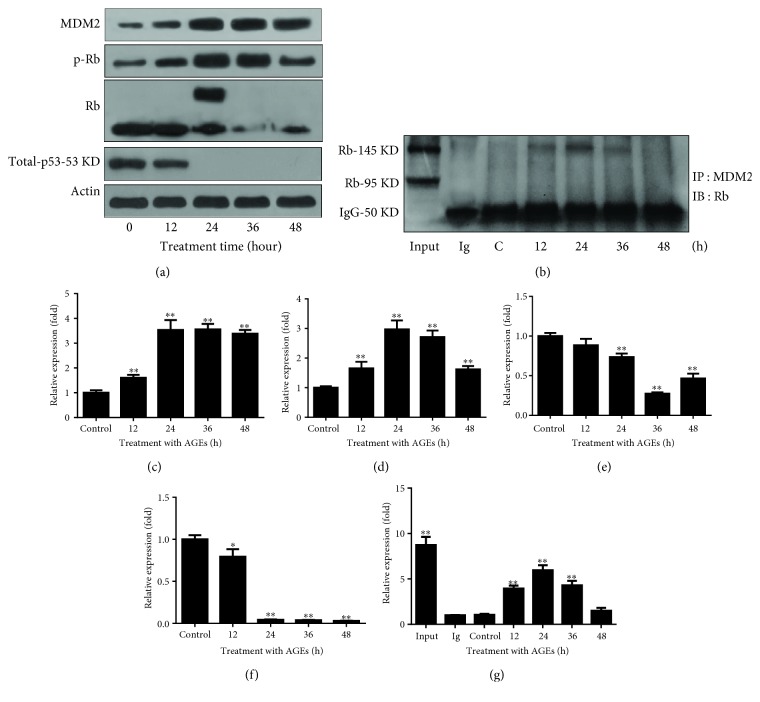
AGEs promote expression of MDM2, degradation of p53 and Rb degradation, and phosphorylation of Rb. (a) Cells were treated with AGEs (50 *μ*g/ml) for indicated times; MDM2, p-Rb, Rb, and p53 were quantified by Western blot analysis, which showed that the expression level of MDM2 was increased and the level of RB as well as p53 was decreased. Phosphorylation of Rb was increased. (b) Cells were treated with BSA-AGEs (50 *μ*g/ml) for indicated times, anti-MDM2 antibody was used for immunoprecipitation, and anti-Rb antibody was used for Western blot analysis, which showed the binding between MDM2 and Rb. Every experiment was repeated at least three times. (c–f) The bands of MDM2 (c), pRb (d), Rb (e), and total p53 (f) in Figure 2(a) were quantified using ImageJ. (g) The band of Rb in Figure 2(b) was quantified using ImageJ. Every experiment was repeated at least three times. Asterisks denote significant difference amongst experimental groups. ^∗^ *p* < 0.05 and ^∗∗^ *p* < 0.01.

Results from the Western blot analyses showed that AGEs upregulated the expression of MDM2 (control (1 ± 0.10): 12 h (1.6 ± 0.12): 24 h (3.5 ± 0.41): 36 h (3.6 ± 0.22): 48 h (3.4 ± 0.15), *p* < 0.01) and downregulated the protein levels of Rb (control (1 ± 0.04): 12 h (0.88 ± 0.08): 24 h (0.74 ± 0.04): 36 h (0.27 ± 0.016): 48 h (0.46 ± 0.06), *p* < 0.01) and p53 (control (1 ± 0.05): 12 h (0.79 ± 0.09): 24 h (0.04 ± 0.004): 36 h (0.038 ± 0.006): 48 h (0.029 ± 0.002), *p* < 0.01) as well as phosphorylation of Rb (control (1 ± 0.05): 12 h (1.6 ± 0.22): 24 h (2.9 ± 0.35): 36 h (2.7 ± 0.22): 48 h (1.6 ± 0.11), *p* < 0.01), which leads to its inactivation, in a time-dependent manner (Figure 2(a)). Moreover, MDM2 directly bound to Rb after 12 (3.9 ± 0.33), 24 (5.9 ± 0.55), and 36 (4.3 ± 0.51) hours of treatment (*p* < 0.01); the maximum binding occurred at 24 hours (Figure 2(b)).

### 3.3. AGE Signals through Its Receptor

**Figure 3:**
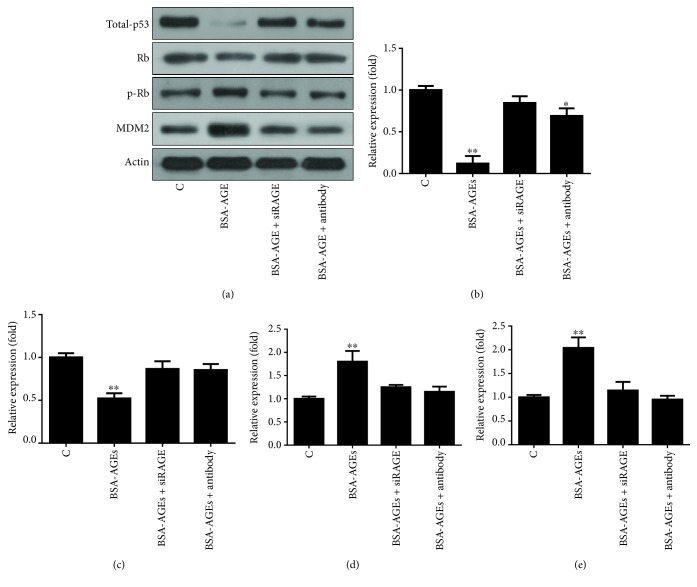
AGEs trigger signaling via their receptor RAGE. Cells were pretreated with RAGE siRNA or anti-RAGE antibody followed by treatment with BSA-AGEs (50 *μ*g/ml) for 24 hours. Both siRNA and anti-RAGE inhibited the effects of AGEs on the levels of MDM2, Rb, and p53. (b–e) The bands of p53 (b), Rb (c), p-Rb (d), and MDM2 (e) in Figure 3(a) were quantified using ImageJ. Every experiment was repeated at least three times. Asterisks denote significant difference amongst experimental groups. ^∗^ *p* < 0.05 and ^∗∗^ *p* < 0.01.

To investigate whether the AGEs bound to its receptor (receptor of advanced glycation end product (RAGE)) to regulate the levels of Rb and p53, we treated the cells with RAGE siRNA that knocked down the RAGE level (Figure 3(a)) or RAGE antibody, which is aimed at inhibiting RAGE signaling, and found that both treatments were able to inhibit the AGEs-caused decrease in protein levels of p53 (control (1.0 ± 0.05): BSA-AGEs (0.12 ± 0.09): BSA-AGEs + siRAGE (0.85 ± 0.08): BSA-AGEs + antibody (0.69 ± 0.09), *p* < 0.01) and Rb (control (1.0 ± 0.05): BSA-AGEs (0.52 ± 0.06): BSA-AGEs + siRAGE (0.87 ± 0.09): BSA-AGEs + antibody (0.85 ± 0.07), *p* < 0.01) as well as the AGE-caused increase in Rb phosphorylation (control (1.0 ± 0.05): BSA-AGEs (1.80 ± 0.23): BSA-AGEs + siRAGE (1.25 ± 0.05): BSA-AGEs + antibody (1.15 ± 0.11), *p* < 0.01) (Figure 3(a)).

### 3.4. KLF5 Transcribes MDM2 in the Cell Model

**Figure 4:**
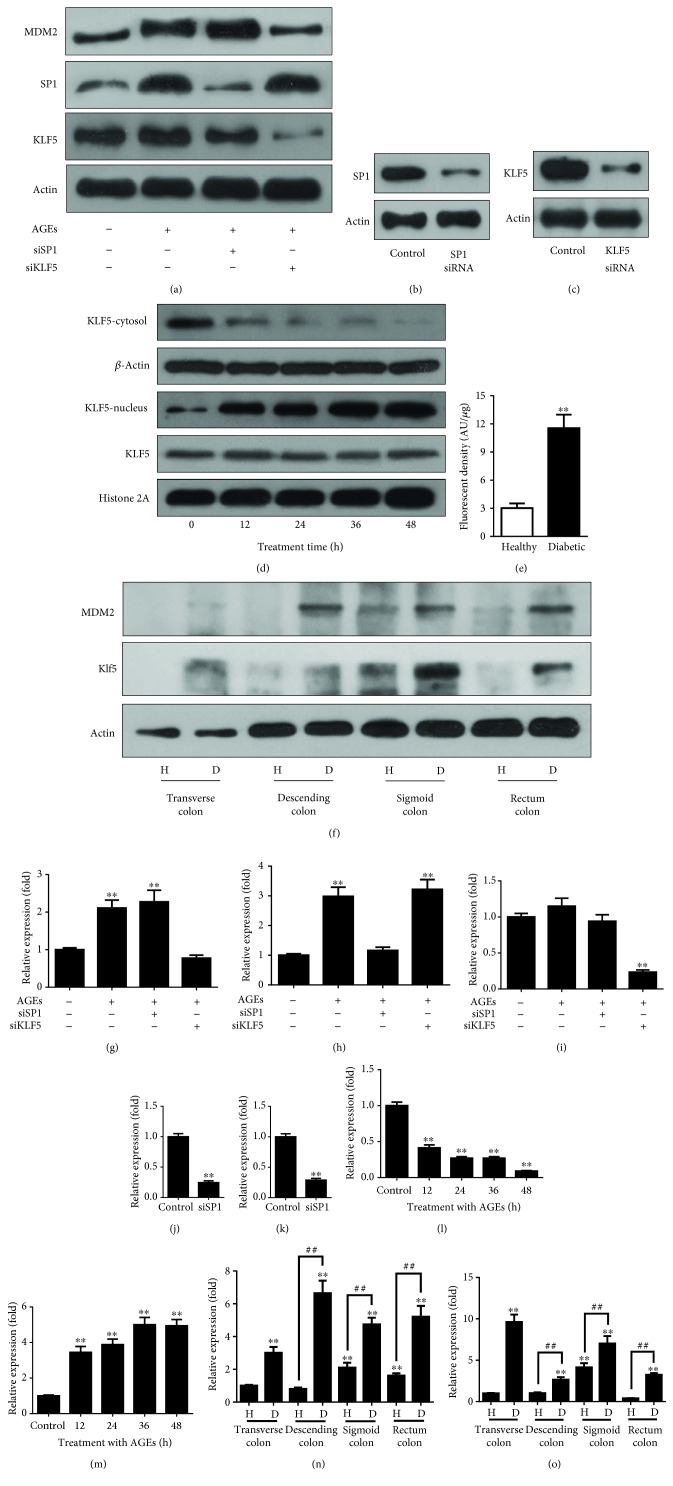
Transcription factor KLF5 regulates MDM2 gene expression. (a) Cells were treated with siRNA target of SP1 or KLF5 to test their effects on the levels of proteins. KLF5 siRNA rather than SP1 siRNA inhibited the AGE-caused changes in the protein levels. (b) SP1 siRNA knocked down the SP1 protein level; (c) KLF5 siRNA knocked down the KLF5 protein level; (d) AGEs triggered the nuclear translocation of KLF5; (e) the level of AGEs in serum of diabetic mice was increased. Serum fluorescent intensity represented the AGE level. (f) The protein levels of both KLF5 and MDM2 were increased in the colon of diabetic mice; (g–i) The bands of MDM2 (g), SP1 (h), and KLF5 (i) in Figure 4(a) were quantified using ImageJ. (j–k) The bands of SP1 (j) and KLF5 (k) in Figures 4(b) and 4(c) were quantified using ImageJ. (l–m) The bands of KLF5 in the cytosol (l) and nucleus (m) in Figure 4(d) were quantified using ImageJ. (n–o) The bands of MDM2 (n) and KLF5 (o) in Figure 4(f) were quantified using ImageJ. Every experiment was repeated at least three times. Asterisks denote significant difference compared to the control group; well number denotes significant difference compared to the healthy group within the same section of colons. ^##^ *p* < 0.01 and ^∗∗^ *p* < 0.01.

We next examined the responsible transcription factor that enhanced the MDM2 expression by targeting at SP1 and KLF5. We found that the treatment increased the protein level of SP1 (1.0 ± 0.05 versus 2.98 ± 0.31, *p* < 0.01) (Figure 4(a)). However, knockdown of SP1 (1.0 ± 0.05 versus 0.28 ± 0.03, *p* < 0.01) (Figure 4(b)) had no effect on the AGEs-enhanced MDM2 expression (Figure 4(a)). Although the protein level of KLF5 was not significantly increased in the AGEs-treated cells (Figure 4(a)), KLF5 siRNA which decreased its protein level (1.0 ± 0.05 versus 0.24 ± 0.03, *p* < 0.01) (Figure 4(c)) was able to abolish the upregulation of MDM2 expression by AGEs (Figure 4(a)). Since the activity of transcription factors is often dependent on subcellular localizations, we investigated the level of KLF5 in the cytosol and nucleus fractions to test whether AGEs increased the nuclear translocation. The results showed that the concentration of KLF5 started to decrease in the cytosol fraction and to translocate to the nucleus from 12 hours (1.0 ± 0.05 versus 0.41 ± 0.04, *p* < 0.01) of the AGEs treatment, which continued to 48 hours (1.0 ± 0.05 versus 0.09 ± 0.007, *p* < 0.01) (Figure 4(d)). We investigated whether KLF5 transcribed the MDM2 animal model of diabetes. Mice were treated with STZ to induce diabetes.  Table 1 shows that the mice became diabetic after 8 weeks of STZ treatment, in particular the AGEs level, as indicated by the increase in fluorescent intensity of serum from diabetic mice (3.0 ± 0.2 versus 11.5 ± 0.7; *p* < 0.01) (Figure 4(e)). The fluorescent intensity was measured at a wavelength of 350 nm which indicated the level of AGEs. The protein level of KLF5 was both increased in the transverse colon (1.0 ± 0.05 versus 3.0 ± 0.35, *p* < 0.01), descending colon (0.8 ± 0.09 versus 6.6 ± 0.77, *p* < 0.01), sigmoid colon (2.1 ± 0.3 versus 4.7 ± 0.41, *p* < 0.01), and rectum colon (1.6 ± 0.14 versus 5.2 ± 0.66, *p* < 0.01) of diabetic mice (Figure 4(f)) as well as the protein level of MDM2 in the transverse colon (1.0 ± 0.05 versus 9.6 ± 0.9, *p* < 0.01), descending colon (1.0 ± 0.1 versus 2.6 ± 0.3, *p* < 0.01), sigmoid colon (4.1 ± 0.5 versus 7.0 ± 0.6, *p* < 0.01), and rectum colon (0.39 ± 0.04 versus 3.2 ± 0.2, *p* < 0.01) of diabetic mice.

### 3.5. Evidence that KLF5 Transcribes MDM2 in an Animal Model of Diabetes

**Figure 5:**
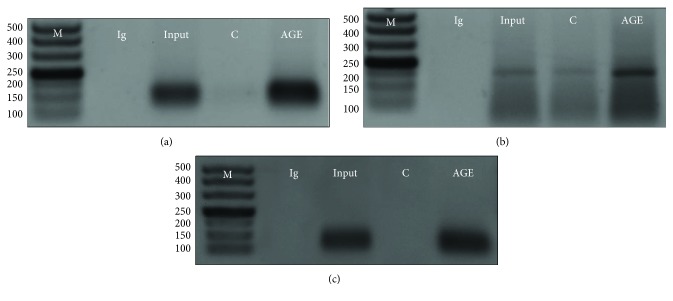
Evidence that KLF5 transcribed MDM2 *in vitro* and *in vivo* in a diabetic animal model. (a) Chromatin immunoprecipitation pulled down the first piece of KLF5-binding sequence (predicted 138 bp) in the human MDM2 gene. (b) Chromatin immunoprecipitation pulled down the third piece of the KLF5-binding sequence (predicted 166 bp) in the human MDM2 gene. (c) Chromatin immunoprecipitation pulled down the third and fourth KLF5-binding sequences in the mouse MDM2 gene, which was predicted to be 190 bp in size; H: healthy; D: diabetic. M: DNA size marker. Every experiment was repeated at least three times.

To obtain further evidence that KLF5 transcribed MDM2, we performed chromatin immunoprecipitation to pull down the transcriptional regulatory sequence of MDM2 using the KLF5 antibody. A total of 4 pieces of human MDM2 gene-binding candidate sequences were obtained after binding site prediction using the JASPAR database (Figure S1). In particular, we designed primers to cover the binding sequences, and the predicted first and third candidate binding sequences were 138 bp and 166 bp in length, respectively. Indeed, we were able to show that the KLF5 antibody pulled down two pieces of sequences which had the calibrated length close to those of the first and third KLF5-binding sequences (138 and 166 bp, respectively; Figure S1) in the transcription regulatory region of MDM2 (Figures 5(a) and 5(b)).

Finally, four pieces of mouse MDM2 gene candidate binding sequences for KLF5 were identified in the JASPAR database (Figure S2). We designed two sets of primers to amplify the binding sequences. Primer 1 amplified the first two binding sequences, and primer 2 amplified the third and fourth binding sequences. Using the KLF5 antibody, we were able to pull down the third and fourth KLF5 binding sequences from the MDM2 gene in chromatin immunoprecipitation assay, which was about the predicted size of 190 bp (Figure 5(c)).

## 4. Discussion

In this study, for the first time, we showed that AGEs *in vitro* could trigger the overexpression of MDM2 (Figure 2(a)) which directly bond to p53 and Rb, which led to Rb and p53 degradation (Figures 2(a) and 2(b)). Increase in MDM2 expression was also seen in a diabetic mouse model (Figure 4(b)). Furthermore, transcription factor KLF5 was found to directly transcribe MDM2 *in vitro* and *in vivo*, which was responsible for the increased MDM2 expression (Figures 4(e), 4(f), and 5(c)).

In diabetes, raised insulin and insulin-like growth factors stimulate cancer cell proliferation and metastasis. Increases in inflammatory cytokines in diabetes promote tumour development, which include interleukin-6 (IL-6), monocyte chemoattractant protein, plasminogen activator inhibitor-1 (PAI-1), adiponectin, leptin, and tumor necrosis factor-alpha [27]. Due to the increase in cytokines, several prooncogenes are hyperactivated in diabetes. Akt/mTOR is positively associated with diabetes cancer initiation and progression [28]. SIRT1 deacetylates and inactivates p53 and HIF1A, active liver X receptor proteins, peroxisome proliferator-activated receptor *γ*, and NFKB1 to promote cell proliferation [29]; RAS signaling, the excessive activation of which is associated with colon cancer initiation, is reported to promote cancer development in diabetes [30].

In normal cells, p53 are carefully controlled at a low concentration by MDM2 and MDMX, presented MDM2 are required for development and protect cells from cellular stress [31]. However, continued presence of MDM2 is required for cancer initiation [32] and results in sustained regression of tumors [33]. MDM2 is known as the main regulator of Rb and p53 in many human cancers [34]. MDM2 is able to mediate Rb degradation directly [35] or in an ubiquitin-independent manner [36]. MDM2 can directly interact with p53 through the N-terminal regions and promote p53 proteasomal degradation in the cytoplasm [37, 38]. Specifically in colon cancer, increases in MDM2 protein expression and gene amplification are observed [12, 39], which is associated with increased colorectal cancer risk and can be used as a prognostic marker [33]. Knockdown of MDM2 enhances the efficacy of cisplatin-based chemotherapy *in vitro* and *in vivo* [40] as well as radiotherapy [41]. In the present study, AGEs promote MDM2 expression which triggers Rb and p53 inactivation/degradation (Figures 2(a) and 2(b)). p53 function is impaired in 69.4% of colon cancer [42, 43]. Knockout or mutation of p53 *in vitro* or *in vivo* can cause tumorigenesis. Mutation of Rb is involved in cancer initiation in many types of human cancer [44]. Conditional depletion of Rb can disrupt the coordination between DNA replication and mitosis [45], induce missed regulation of pluripotency networks [46], and cause early-stage cancer [47]. In embryo cells with mutant p53, knockout of Rb is sufficient to initiate tumorigenesis [48]. Thus, dysregulation of oncogenes and tumor suppressors is implicated in cancer development, including cancer development in diabetes. Our results, more specifically, support a view that AGEs-caused MDM2 overexpression as well as inactivation of p53 and Rb contribute to the development of colon cancer in type 2 diabetes.

Cell stimulation with lysophosphatidic acid can increase the expression of MDM2; under experimental conditions, knockdown of SP1 but not KLF5 blocks the MDM2 overexpression [49]. This shows that, under experimental conditions, SP1 rather than KLF5 serves as the transcription factor for MDM2, which is different from our results that AGEs increased the MDM2 expression via KLF5 (Figure 4(d)). The difference is likely to be due to that different triggers activate different signaling pathways and/or transcription factors. AGEs is found to activate RAS and ERK1/2 in PC12 and colon cancer cells *in vitro* [50, 51]. In IEC6 cells, overexpression of KRAS upregulates KLF5 [52]. Sphingosine-1-phosphate enhances KLF5 expression through the G(i)-protein-Ras-ERK/p38 pathway in neointimal cells and vascular smooth muscle cells [53], indicating that the KLF5 transcriptional activity is closely related to MAPK signaling.

In HCT116 cells, AGEs did not increase the protein level of KLF5, but promoted the nuclear translocation (Figure 4(d)), whereas, in our diabetic mouse model, KLF5 protein level was increased (Figure 5(b)). This might be due to the different behaviors of KLF5 between nontransformed and cancerous cells. Indeed, in nontumorous cells (i.e., IEC8 and IMCE), overexpression of KLF5 enhances cyclin D expression, cell proliferation, and colony formation. In contrast, overexpression of KLF5 in colon cancer cell shows the opposite effects [54]. In cancer cells, microRNA mir-143 and mir-153 directly inhibit the expression of KLF5 [55, 56].

**Figure 6:**
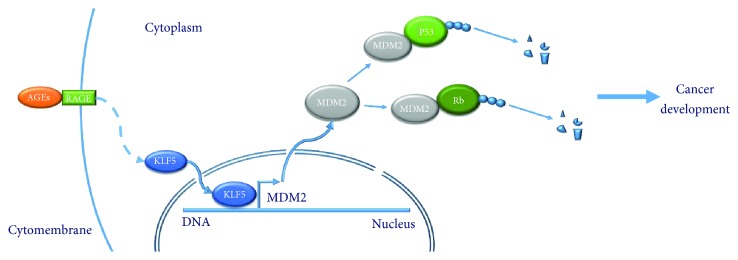
Conclusion figure of our research in this paper. Advanced glycation end products promote the nucleus translocation of transcription factor KLF5, which directly bind to the transcriptional regulatory region of MDM2 and promote its expression. Overexpressed MDM2 directly binds and promotes cancer suppressor Rb and p53 degradation via the ubiquitination pathway.

In conclusion, our results showed that KLF5 directly transcribed MDM2 *in vitro* in a cell model and *in vivo* in diabetes mouse colon, which was responsible for AGEs-increased expression of MDM2 (Figure 6). Overexpression of oncogene (i.e., MDM2) and inactivation of tumor suppressor (i.e., p53 and Rb) are a candidate biological link between type 2 diabetes and colon cancer development.

## Figures and Tables

**Table 1:** Demographic data of experimental animals.

	Healthy subjects	Diabetic subjects
n	6	6
Weight (g)	29.4 ± 2.57	20.8 ± 1.48^∗∗^
Blood glucose (mmol/l)	8.04 ± 1.34	28.7 ± 1.99^∗∗^
HbA1c (mmol/l)	6.55 ± 1.04	17.38 ± 4.64^∗∗^
LDL (mmol/l)	0.12 ± 0.05	0.44 ± 0.27^∗^
HDL (mmol/l)	2.59 ± 0.64	3.536 ± 0.76^∗^
TC (mmol/l)	3.1 ± 1.2	5.12 ± 1.08^∗^
TG (mmol/l)	0.33 ± 0.25	4.58 ± 1.39^∗∗^

Data are means ± SD or medians (range). ^∗^ *p* < 0.05 and ^∗∗^ *p* < 0.01.

## Data Availability

The data used to support the findings of this study are available from the corresponding author upon request.

## Supplementary Materials

Supplementary Materials Figure S1: predicted binding sequences of transcription factor KLF5 in the transcriptional regulatory region of human MDM2 gene and primer design. Figure S2: predicted binding sequences of transcription factor KLF5 in the transcriptional regulatory region of mouse MDM2 gene and primer design.
