# Supplementary material for: Advanced Glycation End Products Increase MDM2 Expression via Transcription Factor KLF5
Source: J Diabetes Res. 2018 Sep 9;2018:3274084. doi: 10.1155/2018/3274084 (PMC6151196; doi:10.1155/2018/3274084)
Supplement: Supplementary Materials — Figure S1: predicted binding sequences of transcription factor KLF5 in the transcriptional regulatory region of human MDM2 gene and primer design. Figure S2: predicted binding sequences of transcription factor KLF5 in the transcriptional regulatory region of mouse MDM2 gene and primer design. [file 3274084.f1.docx]

Fig.S1 Predicted binding sequences of transcription factor KLF5 in the transcriptional regulatory region of human MDM2 gene and primer design.


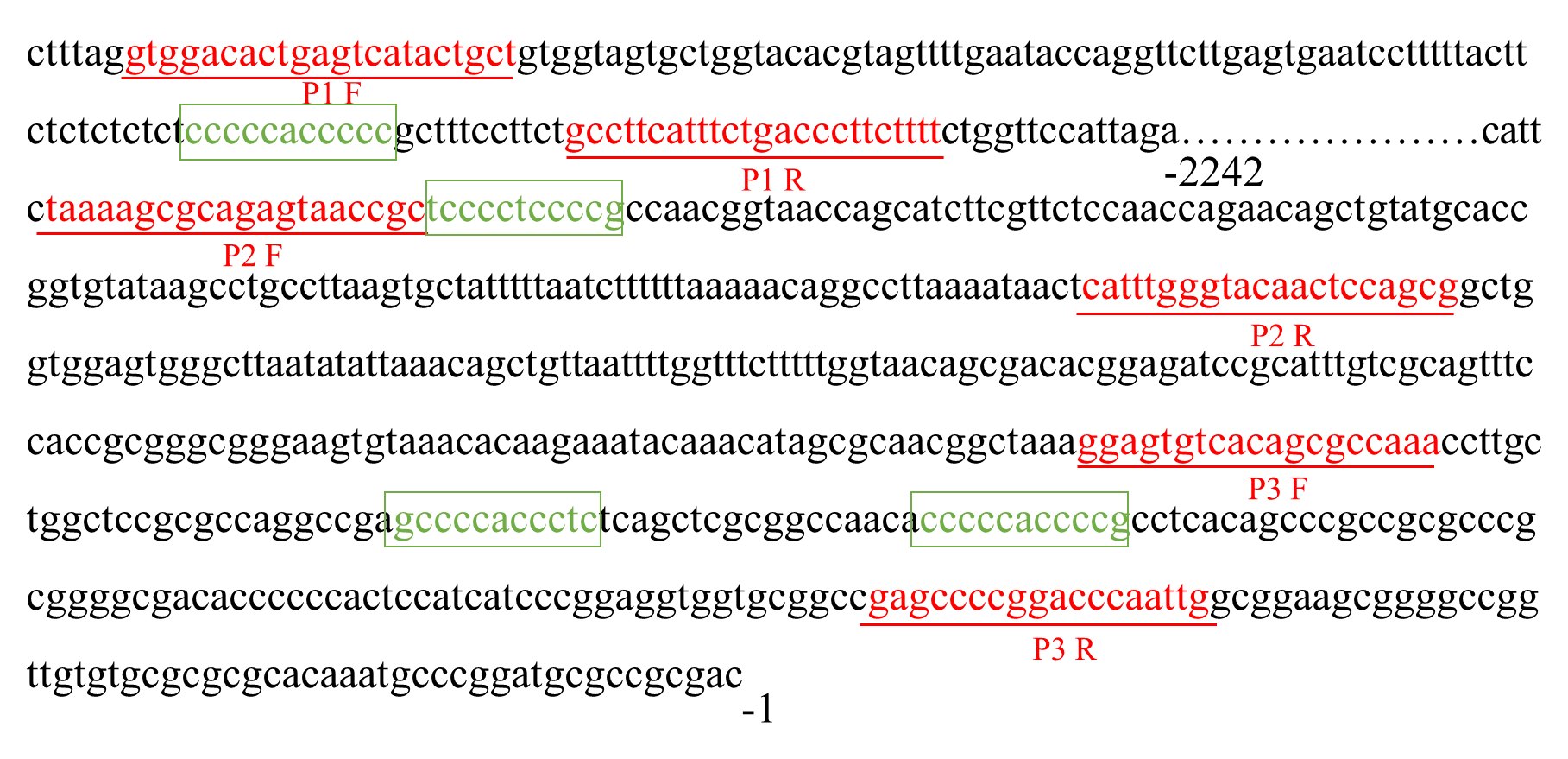


P: primer; F: forward primer; R: reverse primer; MDM2 DNA base in green color in rectangles: predicted KLF5 binding sequences.

Fig.S2 Predicted binding sequences of transcription factor KLF5 in the transcriptional regulatory region of mouse MDM2 gene and primer design


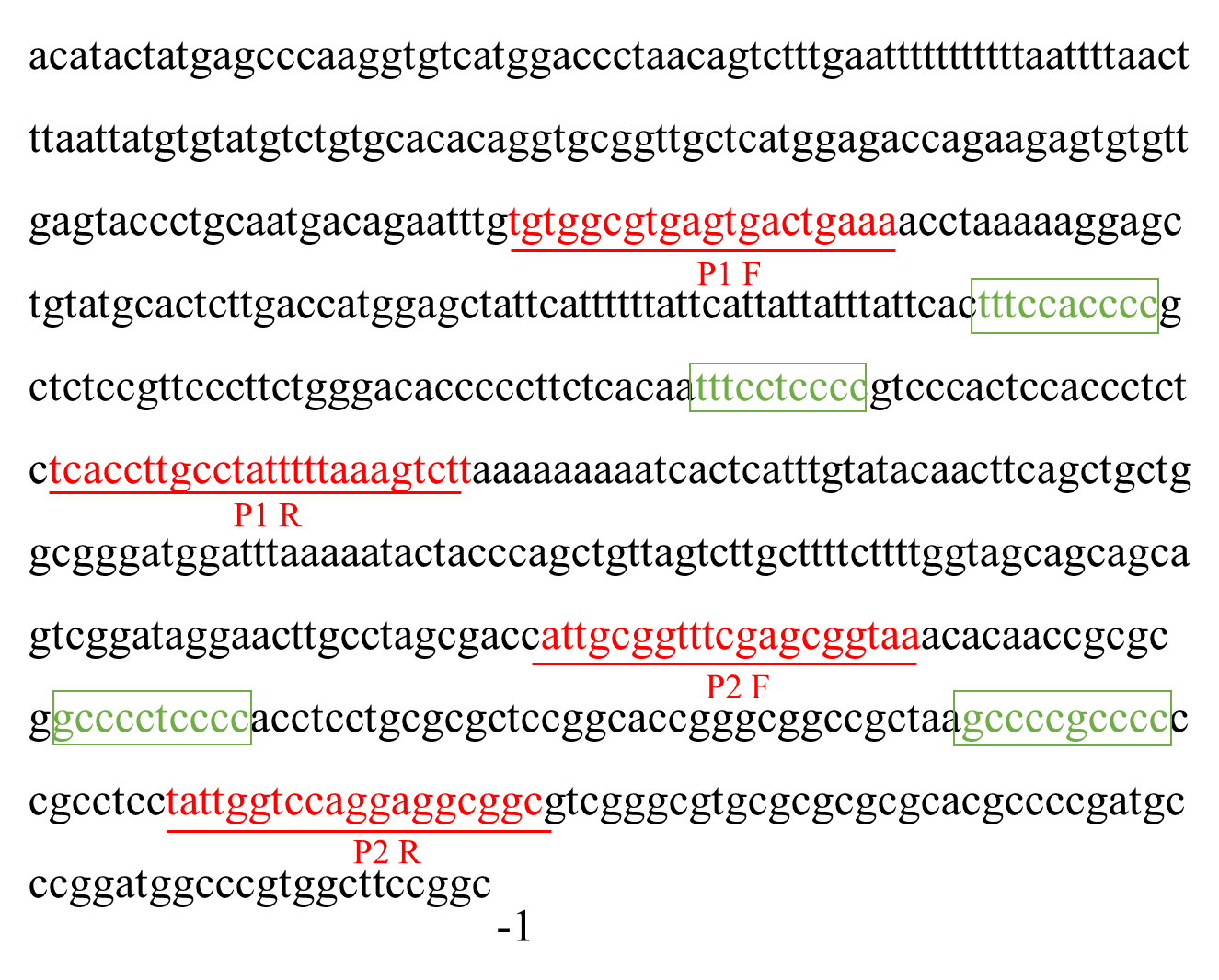


P: primer; F: forward primer; R: reverse primer; MDM2 DNA base in green color in rectangles: predicted KLF5 binding sequences.
